# In vivo hepatic flow distribution by computational fluid dynamics can predict pulmonary flow distribution in patients with Fontan circulation

**DOI:** 10.1038/s41598-023-45396-6

**Published:** 2023-10-24

**Authors:** Petter Frieberg, Pia Sjöberg, Erik Hedström, Marcus Carlsson, Petru Liuba

**Affiliations:** 1 Clinical Physiology, Department of Clinical Sciences Lund, Lund University, Skåne University Hospital, Lund, Sweden; 2 Diagnostic Radiology, Department of Clinical Sciences Lund, Lund University, Skåne University Hospital, Lund, Sweden; 3 Department of Clinical Sciences Lund, Pediatric Heart Center, Lund University, Skåne University Hospital, Lund, Sweden

**Keywords:** Blood flow, Congenital heart defects, Fluid dynamics

## Abstract

In Fontan patients, a lung deprived of hepatic blood may develop pulmonary arterio-venous malformations (PAVMs) resulting in shunting, reduced pulmonary vascular resistance (PVR) and decreased oxygenation. To provide guidance for corrective invasive interventions, we aimed to non-invasively determine how the hepatic to pulmonary blood flow balance correlates with pulmonary flow, PVR, and with oxygen saturation. Magnetic resonance imaging (MRI) data from eighteen Fontan patients (eight females, age 3–14 years) was used to construct patient-specific computational fluid dynamics (CFD) models to calculate the hepatic to pulmonary blood flow. This was correlated with pulmonary vein flow, simulated PVR and oxygen saturation. Clinical applicability of the findings was demonstrated with an interventional patient case. The hepatic to pulmonary blood flow balance correlated with right/left pulmonary vein flow (R^2^ = 0.50), left/right simulated PVR (R^2^ = 0.47), and oxygen saturation at rest (R^2^ = 0.56). In the interventional patient, CFD predictions agreed with post-interventional MRI measurements and with regressions in the cohort. The balance of hepatic blood to the lungs has a continuous effect on PVR and oxygen saturation, even without PAVM diagnosis. MRI combined with CFD may help in planning of surgical and interventional designs affecting the hepatic to pulmonary blood flow balance in Fontan patients.

## Introduction

Congenital heart defects are the most common birth defect, with a reported prevalence of approximately 7 to 10 per 1000 live births^1^. Surgery or catheter-based interventions to reconstruct normal anatomy is used, but when a biventricular repair is not possible, a stepwise univentricular heart palliation leading to a Fontan circulation is now standard^2^. While some children with univentricular hearts fare well, others may develop a wide range of complications such as severe liver fibrosis and cirrhosis, lymphatic congestion, desaturation due to veno-venous collaterals (VVC) or pulmonary arteriovenous malformations (PAVMs) and impaired cardiac output^3^. Complications such as these are often related to elevated central venous pressure and impaired pulmonary hemodynamics. Invasive interventions by catheterization are sometimes required in attempts to optimize flow in the Fontan pathway following surgeries^4, 5^.

**Figure 1:**
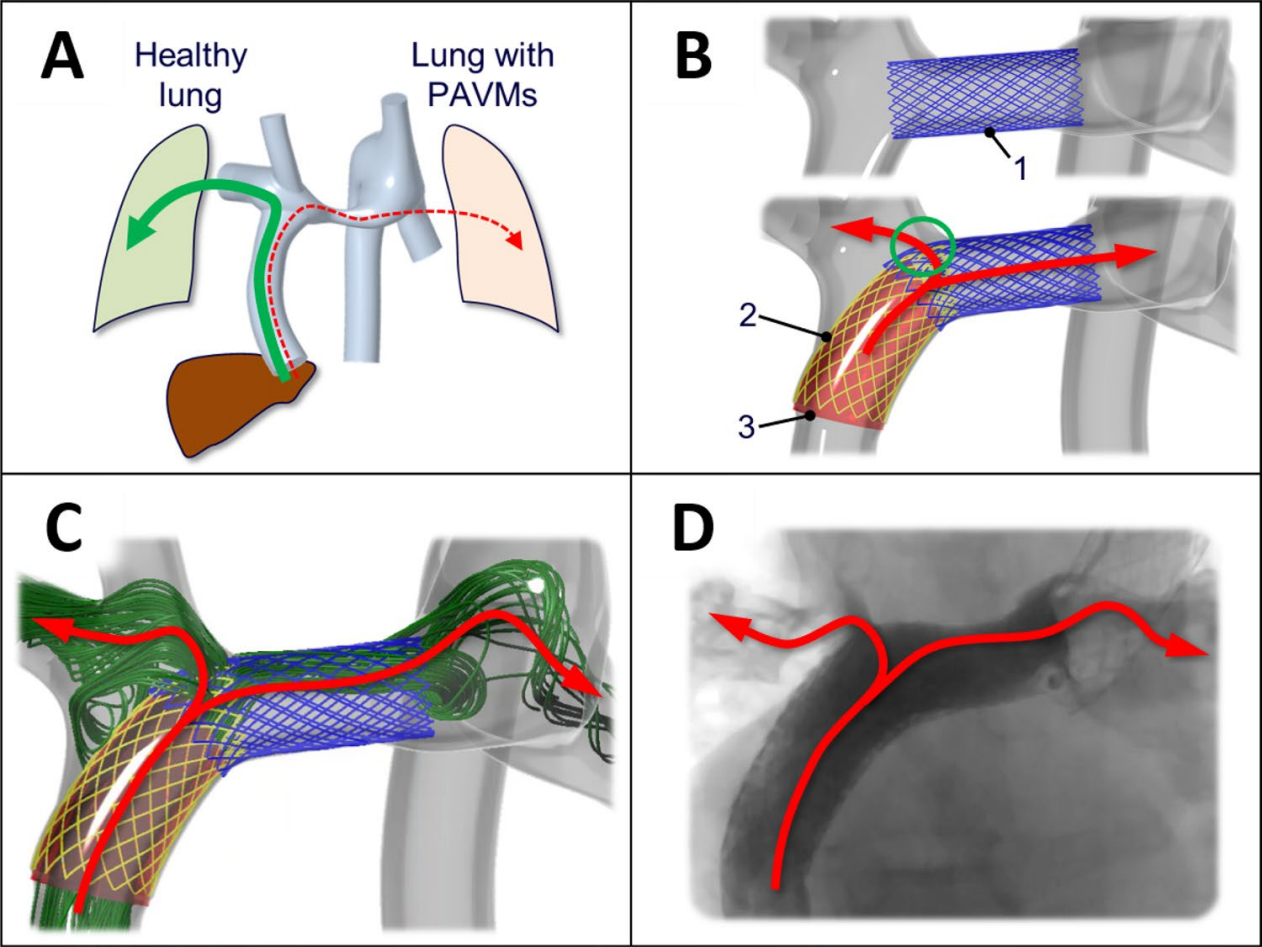
Patient with intervention. (**A**) Patient with hepatic blood flowing mainly to the left right lung and pulmonary arterio-venous malformations (PAVMs) in the left lung. (**B**) Proposed staged endovascular intervention to improve hepatic flow to the left lung. 1: open cell stent in central pulmonary artery. 2–3: open cell stent into conduit with internal covered stent, leaving a fenestration (green circle) for hepatic blood to flow to the right lung. (**C**) Pre-interventional simulation of proposed stenting. (**D**) Post-interventional fluoroscopic angiogram showed good agreement between contrast flow and the pre-interventional simulation.

Furthermore, pulmonary maldistribution of yet unidentified “hepatic factors” may cause pathological PAVMs, which are also observed in the hepatopulmonary syndrome^6–9^. PAVMs are frequently seen in patients with heterotaxy in whom hepatic veins are solely drained via the conduit to the left or right pulmonary artery (Fig. 1A), causing PAVMs in the opposing lung deprived of hepatic factors^10^.

While maldistribution of hepatic factors in the pulmonary circulation has been associated with pathological formation of PAVMs, there is no known quantified relationship between the distribution of hepatic factors and pulmonary resistance due to intrapulmonary shunting.

We hypothesize that even minor pulmonary maldistribution of hepatic blood affects the left and right pulmonary vascular resistance regardless of whether the patient has clinically manifested PAVMs or not.

Magnetic resonance imaging (MRI) is routinely utilized to non-invasively assess anatomy, cardiac function and to measure flow^11–13^. In this context, computational fluid dynamics (CFD) simulations have been shown to provide additional clinical value by enabling in-silico simulations of Fontan pulmonary flow that can predict interventional results^14, 15^. Although long calculation times of up to several hours and associated resources required previously limited use^14, 16, 17^, a recent study showed how calculation times can be reduced to minutes, potentially making the method more widely available^18^.

Therefore, we aimed to utilize a combination of MRI measurements and CFD simulations to non-invasively quantify the relationship between the hepatic to pulmonary blood flow balance and the left/right balance of PVR, and with oxygen saturation at rest, ultimately aiming to better plan and guide future interventions. We additionally aimed to determine if post-interventional changes in a patient case agreed with findings from the studied cohort.

## Methods

### Study population and MRI

Eighteen patients (8 females) aged between 3 and 14 years (median age 7 years) with total cavo-pulmonary connection (TCPC) who had undergone an MRI examination were retrospectively and prospectively included. Inclusion was approved by the National Ethics Review Board in Sweden, and by the regional Ethics Review board in Lund, Sweden. MRI was done 0.5–13 years (median time 5.8 years) after the TCPC, which in all cases included an extracardiac conduit. Patient characteristics are shown in Table 1.

**Table 1:** Patient characteristics.

Patient	Sex	Diagnosis	BSA (m^2^)	Age at MRI	Cardiac Index (l∙min^-1^·m^-2^)	%LPA (%)	HFD (%)	Saturation at rest (%)
1	F	HLHS	0.89	7	2.2	43	19	94
2	M	TA, HRHS	1.08	12	2.5	39	52	93
3	M	HLHS	1.51	13	1.6	29	45	98
4	M	HLHS	1.34	12	2.5	40	61	95
5	M	HLHS	1.71	15	3.5	48	28	97
6	M	PA, VSD, HRHS	1.15	12	2.5	57	36	90
7	F	PA, TGA	1.60	14	3.0	42	51	93
8	M	MS, VSD	0.61	8	2.6	61	12	95
9 (*)	F	AVSD (**)	1.63	14	4.3	60	7	83
10	M	DILV, PA, VSD	0.66	3	3.0	38	63	94
11	F	PA/IVS	0.59	4	2.8	47	46	99
12	M	HLHS	0.60	3	3.1	47	49	97
13	F	HLHS	0.68	6	2.6	64	7	96
14	F	PA/IVS	0.66	4	2.9	52	25	94
15	F	HLHS	0.67	3	2.6	53	27	97
16	M	(**)	0.98	9	3.6	29	70	81
17	M	DORV, AVSD	0.60	3	2.8	58	30	96
18	F	HLHS	0.64	4	2.6	44	25	97

BSA: body surface area. MRI: magnetic resonance imaging. %LPA: fraction of total pulmonary artery blood to the left pulmonary artery. HFD: fraction of total liver blood to the left pulmonary artery. HLHS: hypoplastic left heart syndrome. (*): patient with stent intervention. (**): HLHS, left isomerism, bilateral Glenn/Kawashima. HRHS: hypoplastic right heart syndrome. TA: tricuspid atresia. PA: pulmonary atresia. MS: mitral stenosis. VSD: ventricle septum defect. AVSD: atrium-ventricle septum defect. DILV: double inlet left ventricle. IVS: intact ventricular septum. DORV: double-outlet right ventricle. AVSD: atrioventricular septum defect.

MRI was performed using a Siemens 1.5T Aera (Siemens Healthineers, Erlangen, Germany). Phase contrast flow images in the aorta, pulmonary arteries, pulmonary and caval veins and azygos/hemiazygos veins as needed were collected. Cine images for function were acquired using a steady-state free precession (SSFP) sequence (typical parameters TR/TE/flip angle: 2.9 ms/1.5 ms/60°, slice thickness 5 mm, in-plane resolution 1.2 mm × 1.2 mm). Two-dimensional phase contrast flow measurements were acquired using a velocity encoded fast field echo sequence (typical parameters TR/TE/flip angle: 10 ms/6.5 ms/15° in-plane resolution 1.2 mm × 1.2 mm). Flow measurements of the aorta, superior vena cava (SVC), inferior vena cava (IVC)/TCPC conduit, pulmonary artery branches and pulmonary veins were acquired using phase contrast velocity encoding, typically with 80 cm s^−1^ and for the aorta 200  cm s^−1^. Image analysis was done using the freely available software Segment (Medviso AB, Lund, Sweden, http://segment.heiberg.se)^19^. Aorto-pulmonary collateral (APC) flow was measured as the difference in flow between pulmonary veins and pulmonary arteries in each lung^13^. No contrast agents were used.

Peripheral oxygen saturation was obtained by bedside pulse oximetry when the patient was admitted for MRI examination.

### Computational modeling

Patient-specific three-dimensional models of the major vessels near the TCPC anastomosis were constructed from MRI on boundary curves of the proximal TCPC vessels obtained from Segment, using the computer aided design tool Creo Parametric (PTC, Boston, MA, USA). In patients with image artifacts on the MRI due to stents, obscured anatomic sections were constructed from fluoroscopic images obtained during stent implantation or from follow up catheterization, as described in previous work^20^. Computational fluid dynamics (CFD) simulations were performed using Simcenter FloEFD for Creo (Siemens EDA, Wilsonville, OR, USA), which is embedded in the user interface of Creo Parametric. Steady-state inflows obtained from MRI were used as inlet boundary conditions. Porous media were applied at the outlets to represent PVR and were adjusted such that pulmonary artery flow in the simulations matched the in-vivo pulmonary artery flows measured by MRI in each patient. Blood from the inferior vena cava via the conduit inlet was assumed to be fully mixed with blood from the liver, and the balance of conduit flow to the pulmonary arteries was recorded.

CFD was used to calculate all results representing the percentage of conduit flow to LPA. All other measurements of flow in this study, such as pulmonary vein- and artery flow were obtained from MRI.

### Non-invasive proxies for the left/right balance of PVR

**Figure 2:**
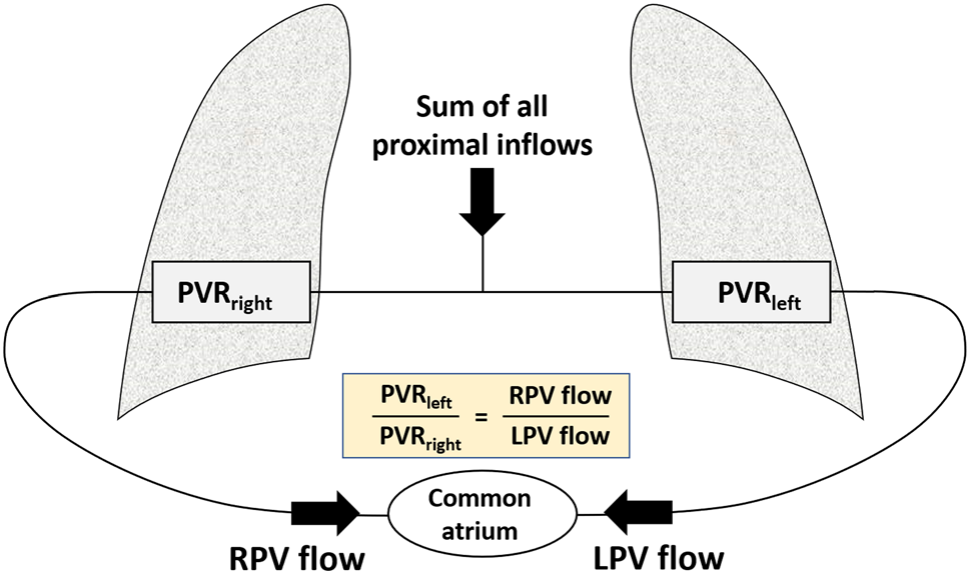
Pulmonary flow. A simplified circuit with pulmonary flow and PVR in the Fontan pulmonary circulation shows how the balance of left/right PVR is proportional to the balance of right/left pulmonary vein flow. RPV: Right pulmonary vein, LPV: Left pulmonary vein. PVR: pulmonary vascular resistance.

In a simplified circuit of the pulmonary circulation, under the assumption that all inflows including collaterals are pre-capillary (Fig. 2), the resulting pulmonary vein flow balance as measured in MRI would be expected to be proportional to the balance of pulmonary vascular resistance of the opposing lungs as shown below.

$$\frac{RPV\;flow}{LPV\;flow} \sim \frac{{PVR}_{left}}{{PVR}_{right}}$$

Therefore, the balance of right/left pulmonary vein flow (RPV and LPV flow respectively) as measured in MRI was used as a non-invasive proxy for the balance of left/right in-vivo PVR. Veno-venous collaterals (VVCs) may also contribute to pulmonary vein flow, therefore an experienced pediatric radiologist reviewed the anatomic MRI images and reported observable VVC’s in the mediastinum.

Additionally, the balance of simulated pulmonary resistance (PVR_left_/PVR_right_) was parametrized and adjusted in CFD such that the pulmonary artery flow in the simulations matched the in-vivo pulmonary artery flows measured by MRI in each patient. Therefore, the balance of left/right simulated pulmonary resistance was also used as a non-invasive proxy for the balance of left/right in-vivo PVR. Further details on the specific methods related to computational modeling, blood rheology and modeling of the balance of PVR is outlined in the Supplementary Material^13, 21^.

### Patient case

The case described in detail below was a 14-year-old patient with heterotaxy, left atrial isomerism, bilateral SVCs, liver veins draining to the common atrium and azygos continuation of the IVC to the left SVC. The patient was palliated with a Kawashima-type procedure at the age of 6 months and an 18 mm conduit between the liver veins and the right SVC at the age of 2 years (Fig. 1A). The patient presented with symptoms of desaturation at rest (81–85%) and significantly impaired exercise intolerance. CT raised suspicion of widespread PAVMs predominantly in the left lower lobe which were demonstrated on cardiac catheterization, presumably due to low conduit inflow to the left lung of hepatic factors from the liver veins. A staged endovascular intervention was suggested to improve the hepatic flow distribution. The patient was referred for pre-interventional MRI and this data was used to perform CFD to guide the planned interventions (Fig. 1C). The three-dimensional CFD model was used to study hemodynamic effects of the fenestration size and location, as well as the central pulmonary artery stent size (blue stent labeled “1” in Fig. 1B). Additionally, the model was used to visualize various combination of stent sizes/lengths to optimize interventional outcome and avoid stent-related complications such as embolization or iatrogenic stenosis. Post-interventional MRIs were performed to evaluate the treatment results at each interventional stage.

### Statistics

Statistical analysis was performed using GraphPad (v9.4.1, La Jolla, CA, USA). Correlations were calculated, and log/linear regressions were used to analyze the relation between the distribution of conduit flow to the pulmonary arteries, the balance of right/left pulmonary vein flow and the simulated balance of left/right pulmonary resistance. Saturation was physiologically expected to be low at the extreme ends of the distribution of conduit flow to the left or right lung, and high in the intermediate range. Therefore, a quadratic regression was chosen to analyze the relation between the balance of conduit flow and saturation. Cohort results were reported as median and range.

### Ethical approval

Retrospective and prospective inclusion was approved by the National Ethics Review Board in Sweden, and by the regional Ethics Review board in Lund, Sweden. Written informed consent was obtained from parents or guardians of all prospectively included patients.

## Results

**Figure 3:**
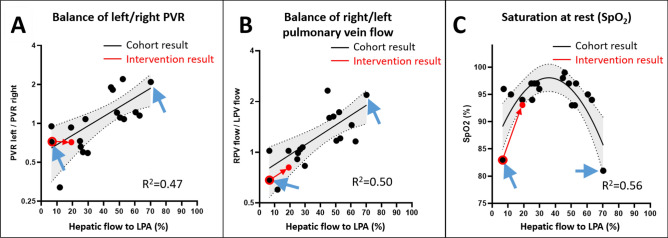
Regressions. (**A**): Regression of the fraction of hepatic flow to the left pulmonary artery (LPA) with the left/right balance of simulated pulmonary vascular resistance (PVR). (**B**): Regression of the fraction of hepatic flow to the left pulmonary artery with the balance of right/left pulmonary vein flow. (**C**): Regression of the fraction of hepatic flow to the left pulmonary artery with saturation at rest (SpO2). Grey area: 95% confidence interval. Red arrows indicate changes in one patient who underwent interventional change that affected the fraction of hepatic flow to the left pulmonary artery. Blue arrows indicate patients diagnosed with pulmonary arteriovenous malformations (PAVMs).

Regression of the balance of simulated left/right PVR with the percentage of conduit flow to the left lung as measured by CFD showed a coefficient of determination R^2^ = 0.47 (Fig. 3A). The median ratio of left to right PVR was 1.08 (0.32–2.2).

Regression of the balance of right/left pulmonary vein flow with the percentage of conduit flow to the left lung as measured by CFD showed a coefficient of determination R^2^ = 0.50 (Fig. 3B). The median ratio of right to left pulmonary vein flow was 1.11 (0.60–2.3).

Regression of the saturation at rest with the percentage of conduit flow to the left lung as measured by CFD showed a coefficient of determination R^2^ = 0.56 (Fig. 3C). Exclusion of the extreme values resulted in a coefficient of determination R^2^ = 0.15.

The median conduit flow percentage to LPA as measured by MRI was 37% (6.6–70%). Observable VVCs in the mediastinum were found in seven patients, of which two patients had VVCs that connected with the pulmonary vein system.

Two patients were previously diagnosed with PAVMs (blue arrows in Fig. 3).

Results as shown in Fig. 3 suggest that with dominant hepatic flow to LPA, the RPV flow is much higher than the LPV flow. Vice versa, with dominant hepatic flow to RPA, the RPV flow is much lower than the LPV flow. Altogether these observations of the distribution of pulmonary flow results suggest lower pulmonary vascular resistance in a lung with low hepatic inflow, with a linear relationship from one end of the hepatic flow spectrum to the other.

### Interventions and results in a patient with PAVMs

Pre-interventional MRI showed very small right-to-left flow in the central pulmonary artery (PA), where CFD showed approximately 7% hepatic flow to LPA. Catheterization showed normal PA pressures, normal transpulmonary gradient and widespread PAVMs in the left lung, particularly in its lower lobe.

In the first of two planned and staged interventions, an open cell stent (36mm IntraStent LD Max, Medtronic, Ireland) mounted on a 14 × 4 BIB balloon was used to dilate the hypoplastic central PA from 8 to 14 mm (Fig. 1B, top). CFD based on pre-interventional MRI showed that hepatic flow to LPA would increase from 7 to 9%. CFD based on post-interventional MRI showed a resulting 8% hepatic flow to LPA.

In the second stage intervention after 6 months, an open cell stent (43mm AndraStent XXL, AndraMed, Germany) mounted on a 20 × 4 BIB balloon was introduced via the left SVC and inflated as scaffolding between the conduit and the right side of the previously inserted stent. A covered graft stent (20 × 48 BeGraft Aortic, Bentley InnoMed, Germany) was introduced via the left SVC through the central PA and into the conduit and was then inflated in a position to leave a fenestration at the superior end for hepatic blood to escape to the right PA (Fig. 1B, bottom). The size of the gap was pre-interventionally tuned in CFD to achieve a hepatic flow to LPA of 20% (Fig. 1C). Pigtail angiography in the conduit showed significant flow through the central PA to LPA (Fig. 1D, right). There were no intraprocedural complications in either of the interventions.

Post-interventional MRI with CFD analysis after 12 months showed that the hepatic flow distribution to LPA increased from 8 to 20%, as predicted by pre-interventional CFD. Cardiac index reduced from 4.3 l/min/m^2^ pre-interventionally with no stents to 3.8 l/min/m^2^ at post-interventional MRI. The right/left pulmonary vein flow balance changed from 0.64 pre-interventionally to 0.75 post-interventionally (Fig. 3B). Pre-interventional MRI showed minor APC to the right lung, which were not seen post-interventionally. There were no APC seen in the left lung pre- or post-interventionally.

In the patient, the simulated left/right PVR balance changed from 0.72 to 0.69 (red arrow in Fig. 3A). In the patient, saturation at rest increased from approximately 83% to 93%, or an absolute increase of 10% (red arrow in Fig. 3C).

Additionally, the patient reported improved clinical status including exercise tolerance.

Computational fluid dynamics run-times were less than 10 min per simulation. CFD simulations were performed during the final intervention to evaluate effects of creating a fenestration in the covered stent (Fig. 1B,C), which was found to be unfavorable due to risk of potential metal filament rupture.

## Discussion

In this study we have for the first time shown a continuous relationship between hepatic flow distribution to the lungs and MRI-based PVR assessment. These results imply that the pulmonary distribution of hepatic blood is associated with a continuous distribution of PVR, and therefore that the hepatic flow distribution has a subclinical effect on the pulmonary vasculature even in the absence of visually detectable PAVMs.

### Clinical significance

The pulmonary distribution of hepatic blood from the conduit correlated well with oxygen saturation, with the distribution of pulmonary vein flow and with pulmonary resistance as calculated by CFD. This means that if CFD is used to predict changes following interventions aimed at changing the hepatic blood flow distribution to the lungs, the same method can also be used to predict post-interventional changes in pulmonary vein flow, changes in pulmonary resistance and changes in oxygen saturation at rest.

Catheter-based interventions are less invasive and provide the benefit of avoiding sternotomy in patients with at least 2–3 earlier sternotomies. As a practical example in the studied interventional patient, the post-interventional changes in pulmonary vein flow and oxygen saturation followed the regression curves near the confidence interval obtained from the studied cohort.

While we recognize that few centers currently have routine access to CFD to characterize the distribution of intrinsic pulmonary resistance as demonstrated here, the results also indicate that measurements of pulmonary vein flow distribution as measured using more broadly available MRI also correlate well with the distribution of hepatic blood via the conduit. This means that MRI measurements of pulmonary vein flow may be utilized to screen for changes in the pulmonary vasculature in patients suspected of developing both subclinical and fulminant PAVMs due to pulmonary maldistribution of hepatic blood.

Of note, the median ratio of right to left pulmonary vein flow in the patients was 1.11. In healthy adults, the right to left pulmonary vein flow distribution is approximately 1.07, calculated from the percentage of total pulmonary vein flow to the left pulmonary veins of 48 ± 1% as reported by Wieslander et al.^22^. The pulmonary vein flow ratio of 1.11 was observed even in the presence of a wide range of APC (up to 31% of cardiac output). This suggests that APC flow compensates for imbalances in the proximal pulmonary artery flow such that the pulmonary vein flow distribution is normalized. Additionally, this implicitly means that the distribution of PVR in patients is on average the same as in healthy adults.

### Modeling approaches

In this project the previously described “lean” CFD modeling based on time averaged, non-invasive MRI measurements and a linear approximation of PVR^18^ was successfully utilized to devise and predict the outcome of a complex endovascular intervention in a patient with PAVMs. The pre-interventionally performed simulations were similar to the observed post-interventional flow distribution as measured in MRI and were also visually similar to the fluoroscopic angiogram obtained during the intervention (Fig. 1D). Notably, complementary simulations were also performed during the on-going final intervention (Fig. 1C,D) to evaluate results based on hemodynamic findings as they occurred, demonstrating the potential of lean and clinically integrated CFD to guide treatment during interventions.

### Calculation of the hepatic flow distribution

The hepatic flow distribution was in this work calculated in CFD under the assumption that blood from the liver veins is completely mixed with blood from the IVC in the conduit. Rijnberg et al*.* showed in a study of fifteen patients that blood may not be completely mixed during its course in the conduit to the pulmonary arteries, as calculated by particle tracing using 4D flow MRI^23^. Since 4D flow MRI was not used in this work, we therefore believe that improved hepatic flow tracing using 4D flow MRI in future studies may strengthen the findings of the current study and provide data for validation reasons.

### Limitations

Veno-venous collateral contributions to the pulmonary vein flows measured in MRI is a confounder in the described relationship between pulmonary vein flow distribution and pulmonary resistance distribution. In this study, visually observable VVCs in the mediastinum were however found in less than half of the patients. Drainage to the pulmonary vein system was only observed in two patients, but the flows could not be quantified in MRI, indicating that VVCs only had a minor contribution to the pulmonary vein flow as measured in the studied patients.

Measurements of peripheral oxygen saturation can differ at different times in the same patient. While we found a strong correlation between oxygen saturation and HFD, with physiologically expected low oxygenation at the extreme ends of the HFD range, the correlation is likely less accurate in the middle ranges of HFD.

## Conclusion

In summary, we have quantified the relationship between the pulmonary distribution of hepatic blood, pulmonary vein flow distribution and pulmonary resistance distribution, and have additionally demonstrated the clinical utility of a fast and clinically integrated CFD method to predict outcome of an intervention in a patient with PAVMs. These findings may help patients by improving patient-specific predictions on how different conduit designs may affect the formation of PAVMs.

### Supplementary Information

Supplementary Information.

## Data Availability

No data from external sources were used. The authors have access to all data.
