# Supplementary material for: In vivo hepatic flow distribution by computational fluid dynamics can predict pulmonary flow distribution in patients with Fontan circulation
Source: Sci Rep. 2023 Oct 24;13:18206. doi: 10.1038/s41598-023-45396-6 (PMC10598063; doi:10.1038/s41598-023-45396-6)
Supplement: Supplementary file 1 — Supplementary Information. [file 41598_2023_45396_MOESM1_ESM.pdf]

Supplementary material

Title

In vivo hepatic flow distribution by computational fluid dynamics can predict pulmonary flow distribution in patients with Fontan circulation

Authors

* Petter Frieberg MD PhD MSc[1], Pia Sjöberg MD PhD[1], Erik Hedström MD PhD[1,2], Marcus Carlsson MD PhD[1] , Petru Liuba MD PhD[3]

[1] Clinical Physiology, Department of Clinical Sciences Lund, Lund University, Skåne University Hospital, Lund, Sweden

[2] Diagnostic Radiology, Department of Clinical Sciences Lund, Lund University, Skåne University Hospital, Lund, Sweden.

[3] Pediatric Heart Center, Department of Clinical Sciences Lund, Lund University, Skåne University Hospital, Lund, Sweden

Computational modeling of the distribution of PVR

The left and right intrinsic pulmonary resistances (PVR_{left} and PVR_{right}) were parametrized with a ratio factor c such that:

$$PVR_{left} = c \cdot PVR_{right} \qquad (1)$$

Physiological values of the total PVR (PVR_{tot}) for both lungs were used with the ratio factor c such that:

$$PVR_{left} = PVR_{tot} \cdot (c + 1) \qquad (2)$$

$$PVR_{right} = PVR_{tot} \cdot \frac{(c + 1)}{c} \qquad (3)$$

which together satisfy the fundamental relation between total PVR and left and right PVR:

$$\frac{1}{PVR_{tot}} = \frac{1}{PVR_{left}} + \frac{1}{PVR_{right}} \qquad (4)$$

This distribution of the intrinsic pulmonary resistances was used together with adjustments for the presence of APC as described next. PVR_{tot} was uniformly set to 3 Wood Units (WU).

Modeling the effect of APC inflow as an increase of the intrinsic PVR in each lung

It is commonly assumed that APCs are mainly precapillary and perfuse the distal pulmonary artery vasculature, where saturation step-up from SVC / conduit-IVC to distal branch PAs supports this assumption. APC inflow can be quantified indirectly with CMR as the difference between pulmonary vein flow (*PV flow*) and pulmonary artery flow (*PA flow*) in each lung (1). VVCs may also contribute to pulmonary vein flow, therefore an experienced pediatric radiologist reviewed the anatomic CMR images and reported observable VVC's in the mediastinum.

With these considerations, it follows that in the presence of APC, proximal pulmonary artery flow is less than the pulmonary vein flow in each lung respectively. Instead of physically introducing APC flow as an additional inlet into the CFD simulation model, the effect of APC inflow was modeled as an increase of the intrinsic PVR in each lung as outlined next.

Due to APC inflow, the total pulmonary vein flow is greater than the proximal pulmonary artery flow with a factor:

$$\frac{PV\ flow}{PA\ flow} \tag{5}$$

which can be obtained from CMR for each patient and for each lung. By artificially increasing the intrinsic pulmonary vascular resistance (PVR_{left} and PVR_{right} respectively) with this flow-factor, the internal flow in the TCPC will be identical to a solution where APC flow was physically introduced in the distal pulmonary artery.

In summary, using this flow-factor as a proxy of the presence of APC in combination with the previously outlined parametrization c of the intrinsic PVR for each lung, the total PVR for each lung was modeled as shown in Figure 1A and according to:

$$PVR_{tot,left} = PVR_{tot} \cdot (c+1) \cdot \frac{LPV\ flow}{LPA\ flow} \tag{6}$$

$$PVR_{tot,right} = PVR_{tot} \cdot (c+1) \cdot \frac{RPV\ flow}{RPA\ flow} \tag{7}$$

These resistances were applied as linear porosities to thin baffles near the pulmonary artery outlets of the CFD model (Figure 1B). In patients where multiple pulmonary artery branches were visualized and modeled, the total resistance was distributed inversely proportional to the vessel cross section area fraction. CFD simulations were then iterated to find a patient-specific ratio factor c such that simulation results matched CMR results in terms of the observed, patient-specific proximal pulmonary artery flow distribution. CFD was used to measure the fraction of hepatic blood to the left pulmonary artery. Thus, by obtaining the factor c from the simulations, this approach allowed the study of the relation between hepatic flow distribution and the distribution of the intrinsic pulmonary resistances (PVR_{left} and PVR_{right}).

Simulations were performed under the assumptions of rigid vessel walls, laminar flow, constant atrial pressure, and patient-specific steady-state inlet flows obtained from CMR at each inlet.

Linear pulmonary resistance was simulated using linear porosity applied to 0.5 mm thick baffles placed proximal to the outlets (Figure 1B). Blood viscosity η(γ) was defined as an incompressible non-Newtonian fluid based on the Carreau model according to equation (8) with density=1060 $kg \cdot m^{-3}$, zero shear viscosity η0=0.033 $kg \cdot m^{-1} \cdot s^{-1}$, infinite shear viscosity η_∞=0.006109 $kg \cdot m^{-1} \cdot s^{-1}$, time constant λ=3.34 s, and power law index n=0.3035 (2).

$$\eta(\gamma) = \eta_\infty + (\eta_0 - \eta_\infty)[1 + (\gamma\lambda)^2]^{\frac{n-1}{2}} \tag{8}$$

All 3D modeling and CFD simulations were prepared on a standard DELL XPS 15 laptop with an Intel i7-7700HQ processor and 16 GB RAM and an NVIDIA GTX 1050 graphics card. The graphics card does not contribute to the CFD flow calculations.

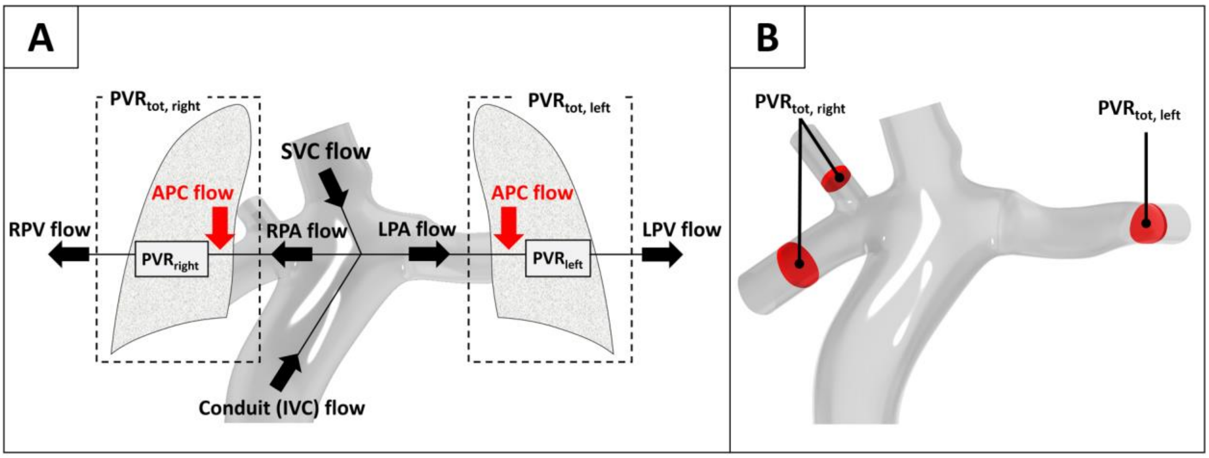

Figure 1. **Panel A:** Schematic diagram of pulmonary flow and resistance in a Fontan patient. *SVC:* superior vena cava. *IVC:* inferior vena cava. *LPA:* left pulmonary artery. *RPA:* right pulmonary artery. *RPV:* right pulmonary vein. *LPV:* left pulmonary vein. *APC:* aorto-pulmonary collaterals. *PVR:* pulmonary vascular resistance. **Panel B:** Placement of porous baffles used to simulate PVR in computer simulations.

1. Grosse-Wortmann L, Al-Otay A, Yoo SJ. Aortopulmonary collaterals after bidirectional cavopulmonary connection or fontan completion quantification with MRI. Circ Cardiovasc Imaging. 2009;2(3):219–25.
2. Wei Z, Singh-Gryzbon S, Trusty PM, Huddleston C, Zhang Y, Fogel MA, et al. Non-Newtonian Effects on Patient-Specific Modeling of Fontan Hemodynamics. Ann Biomed Eng. 2020;48(8):2204–17.
